# Nanostructured surfaces by supramolecular self-assembly of linear oligosilsesquioxanes with biocompatible side groups

**DOI:** 10.3762/bjnano.6.244

**Published:** 2015-12-11

**Authors:** Maria Nowacka, Anna Kowalewska, Tomasz Makowski

**Affiliations:** 1 Centre of Molecular and Macromolecular Studies, Polish Academy of Sciences, Sienkiewicza 112, 90-363 Łódź, Poland

**Keywords:** atomic force microscopy, hydrophilicity, oligosilsesquioxanes, self-assembly, surface

## Abstract

Linear oligomeric silsesquioxanes with polar side moieties (e.g., carboxylic groups and derivatives of *N*-acetylcysteine, cysteine hydrochloride or glutathione) can form specific, self-assembled nanostructures when deposited on mica by dip coating. The mechanism of adsorption is based on molecule-to-substrate interactions between carboxylic groups and mica. Intermolecular cross-linking by hydrogen bonds was also observed due to the donor–acceptor character of the functional groups. The texture of supramolecular nanostructures formed by the studied materials on mica was analysed with atomic force microscopy and their specific surface energy was estimated by contact angle measurements. Significant differences in the surface roughness, thickness and the arrangement of macromolecules were noted depending on the kind of functional groups on the side chains. Specific changes in the morphology of the surface layer were observed when mica was primed with a monolayer of small organic compounds (e.g., *N*-acetylcysteine, citric acid, thioglycolic or acid). The adsorption of both silsesquioxane oligomers and organic primers was confirmed with attenuated total reflectance infrared spectroscopy. The observed physiochemical and textural variations in the adsorbed materials correlate with the differences in the chemical structure of the applied oligomers and primers.

## Introduction

The modification of surface properties can be used as a versatile tool in materials engineering for biological and medical purposes [1–4]. The focus has been more recently shifted towards hydrophilic surfaces due to their antifouling properties [5]. Cell biology applications also require new materials that could mimic the natural biological environment of cells and resemble the natural extracellular matrix (ECM). The surface chemical composition and topography that define the free energy [6–9] also impact the pre-adsorbed protein layer and can mediate cell–substrate interactions [3,10–21]. Substrates bearing COOH groups can be used to control the cell behaviour via interactions with the underlying matrix. For example, surfaces carrying COOH groups were applied for studies on the effect of surface wettability on protein adsorption and adhesion of human umbilical vein endothelial cells (HUVECs) and HeLa cells [3], human fibroblasts [14], human mesenchymal stem cells [15,22], corneal epithelial cells [23], fibroblasts [24], myoblasts [25] and endothelial cells [26]. Substrates with COOH groups were also used to elucidate the role of chemistry-dependent differences in cell differentiation owing to specific binding to proteins adsorbed on the surface [25,27–28].

Well-defined substrates made of small molecule self-assembled monolayers (SAMs) [3,14,23–30] and self-assembled polymer monolayers (PSAMs) [12,31–33] are thus promising candidates for such purpose-tailored bioengineering tools. The structure of the SAMs and PSAMs strongly depends on the operating mechanism of adsorption [34–35]. These two types of monolayers can differ significantly due to conformational variabilities observed for macromolecular chains. On the other hand, PSAMs offer improved surface stability, ease in processing, unique chemical specificity and tunable surface energy [12,31–33,36].

We have recently found that linear oligosilsesquioxanes functionalized with 2-(carboxymethylthio)ethyl side groups (LPSQ-COOH) can adsorb from their solutions and spontaneously form well-ordered and stable, PSAM-type, 2D nanolayers at the surface of muscovite mica, which renders the surface exceptionally hydrophilic [37–38]. Muscovite mica, chosen as a substrate for the present study, is a layered aluminosilicate [KAl_2_(Si_3_AlO_10_)(OH)_2_] that exhibits interesting surface properties and chemical specificity. Potassium ions electrostatically bind the alternating aluminosilicate sheets in the lamellar structure of mica. The mineral can be easily cleaved along the plane located in the K^+^ layer to expose a perfectly smooth surface [39] that can serve as a very good AFM imaging substrate for studies on biomaterials [40–41] and polymers [42–43]. Upon exfoliation, K^+^ becomes accessible to acidic molecules and can be involved in the formation of surface salts. For example, potassium carboxylates generated on the surface of mica assist the process of adsorption of fatty acids [44–47] and their derivatives [48].

The character of the interactions between the oligomers and the substrate also defines the structure of the assemblies of LPSQ-COOH on mica [37]. It was thus of interest to study if the morphology of the surface layer of PSAMs and its physicochemical properties can be changed by alteration of the mechanism of adsorption on mica. The structure of the PSAMs was engineered both by alteration of the functional groups on the surface as well as those belonging to the side chains of LPSQ. In this report we present the modification of mica with linear oligomeric silsesquioxanes (LPSQ-COOH/X) with side groups bearing 2-(carboxymethylthio)ethyl where the X-groups are derivatives of *N*-acetylcysteine (NAC), cysteine hydrochloride (Cys-HCl) and glutathione (GSH). Such self-assembled PSAMs based on polysilsesquioxane materials are attractive for surface nanopatterning and bioengineering, including preparation of surfaces rich in organic groups typical of the extracellular matrix in living organisms (e.g., CH_3_, OH, NH_2_ and COOH).

We have investigated the effect of the kind of functional groups in side chains of LPSQ-COOH/X on the structure (e.g., surface roughness, thickness and arrangement of macromolecules within the coated layer) of the prepared PSAMs. Native mica was used bare or primed, prior to the coating with LPSQ-COOH/X, with a monolayer of *N*-acetylcysteine, citric acid or thioglycolic acid. The primers are bound to mica by ionic bonds (carboxylates) and simultaneously provide the substrate with new organic functions capable of hydrogen bonding [49].

Atomic force microscopy (AFM) and attenuated total reflectance infrared spectroscopy (ATR-FTIR) were used as analytic tools for the studies. The changes in the free surface energy of the prepared hydrophilic surfaces were also investigated for all LPSQ-COOH/X adsorbed on native and primed mica. The obtained results suggest that both the composition of side polymer chains and the kind of functional groups on the surface are key factors defining the structure and properties of PSAMs based on LPSQ-COOH/X.

## Results and Discussion

### Supramolecular assemblies of LPSQ-COOH/X on native mica

**Scheme 1:**
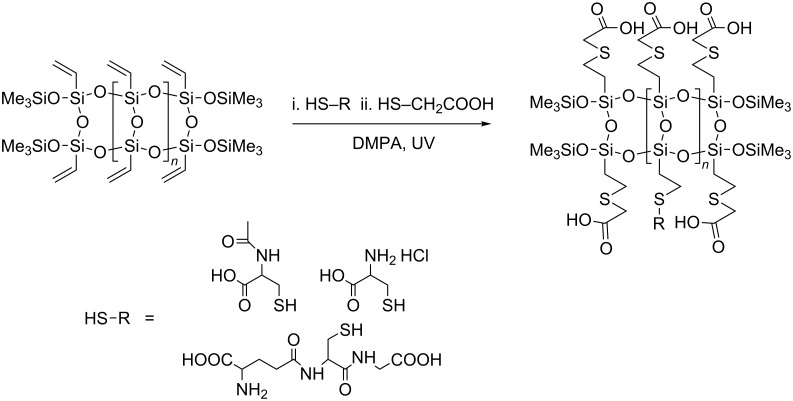
Synthesis of LPSQ-COOH/X.

**Figure 1:**
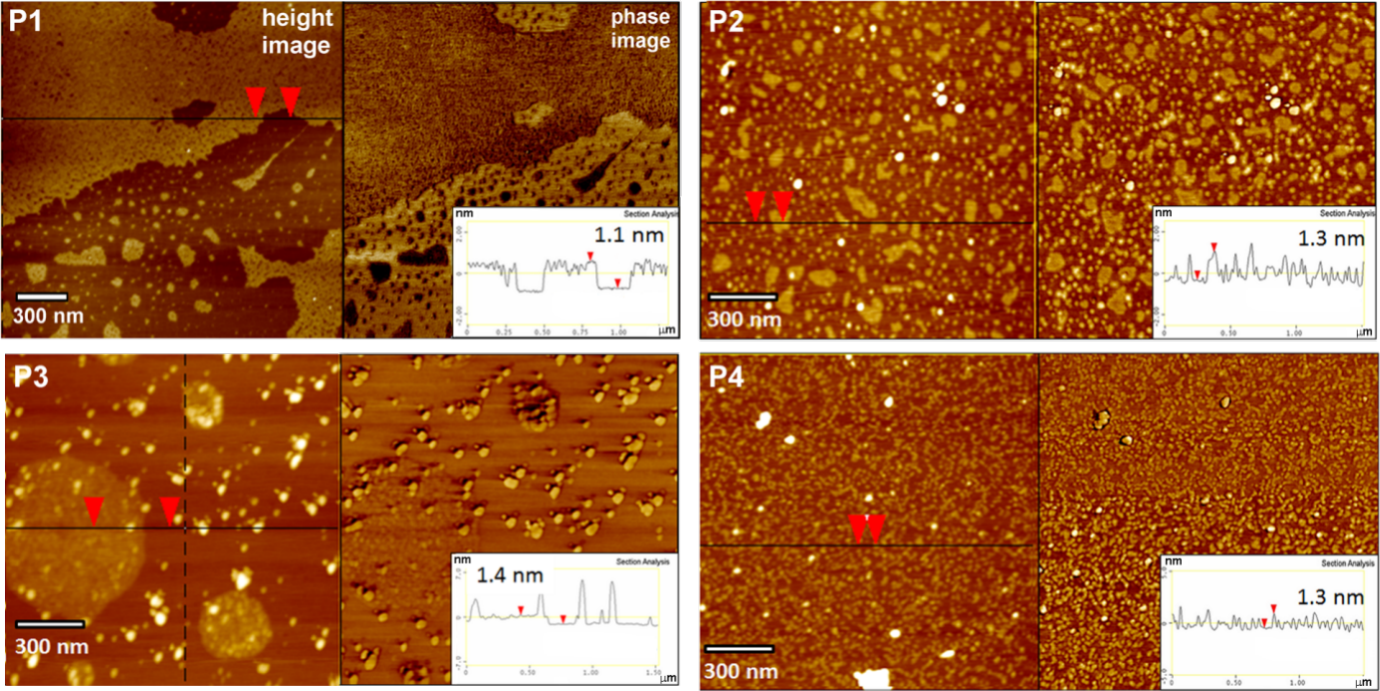
AFM height and phase images and the corresponding surface profiles of P1, P2, P3 and P4, dip-coated on bare mica. P1, P3 and P4: 0.045 wt % solutions in THF; P2: 0.045 wt % solution in MeOH; immersion time *t*_i_ = 5 s.

Functionalized ladder-like silsesquioxanes (LPSQ-COOH/X) were prepared (Scheme 1, Table 1) by the two-step addition of organic thio-derivatives, i.e., thioglycolic acid, *N*-acetylcysteine (NAC), glutathione (GSH) and cysteine hydrochloride (Cys-HCl), to the side chains of vinyl-containing LPSQ precursors obtained by polycondensation of cyclic tetravinylsiloxanetetraols [50]. The thiol-ene additions were photoinitiated by 2,2-dimethoxy-2-phenylacetophenone (DMPA) (full experimental data can be found in Supporting Information File 1). Thin layers of LPSQ-COOH/X were deposited onto freshly cleaved mica substrates by dip coating from their diluted solutions and the morphology of the coated samples was studied with AFM (Figure 1).

**Table 1:** Composition of the prepared LPSQ-COOH/X.

Sample	Side group amount (mol %)			
	COOH	Cys-HCl	GSH	NAC

P1	100	–	–	–
P2	80	20	–	–
P3	80	–	20	–
P4	80	–	–	20

### AFM studies of LPSQ-COOH/X coated on native mica

The structure and reactivity of LPSQ-COOH/X makes the polymers suitable for the formation of planar PSAMs on various reactive surfaces. Side carboxylic groups in LPSQ-COOH/X allow for a very efficient polymer anchoring on the surface due to both multipoint ionic substrate–adsorbate interactions and adsorbate–adsorbate hydrogen bonding [37]. The formation of ordered SAMs and PSAMs at the liquid–solid interface can occur only if it is energetically allowed by entropy–enthalpy compensation [51–53]. Thus, the mechanism of adsorption of LPSQ-COOH/X on mica should be discussed with respect to possible intermolecular interactions between polymer chains and their relations with the substrate. Macromolecules consisting of surface-reactive repeating units can made for a special case of PSAMs – the one not anchored at the end point but adsorbed parallel to the surface [31]. The thickness of such PSAMs depends on the flexibility of the polymer backbone and its affinity for the surface [54]. In the case of high-affinity adsorption and rigid polymers, it is close to the chain width.

The results obtained for LPSQ-COOH/X using AFM (topographic images and height profiles) suggest a specific packing of the chains on the surface of mica. LPSQ-COOH can form fine nanolayers (Figure 1) of thickness that correlates well with the estimated macromolecule width. This was estimated to be about 1.6 nm, as calculated for the structure of a LPSQ-COOH oligomer constructed on HyperChem platform and modelled in vacuum using a molecular mechanics force field MM+ method (Polak–Ribiere/conjugate gradient optimization algorithm) and a semi-empirical PM3 method (single point energy calculations [37]. However, macromolecules of LPSQ-COOH/GSH, LPSQ-COOH/Cys-HCl and LPSQ-COOH/NAC do not easily extend parallel to the mica substrate. The surface of coated samples is covered with globular nano-objects (Figure 1) that can be possibly formed by single oligomers (or their clusters) that are coiled due to intramolecular hydrogen bonding between the compatible side groups. ATR-FTIR spectra (Supporting Information File 1) confirmed that COOH groups in all the studied polymers are involved in the formation of adsorbed surface structures (a substantial decrease of the ν_C=O_ band at ≈1700 cm^−1^ and emergence of diffuse bands in the formed PSAMs was observed).

### Surface energy of LPSQ-COOH/X coated on native mica

**Figure 2:**
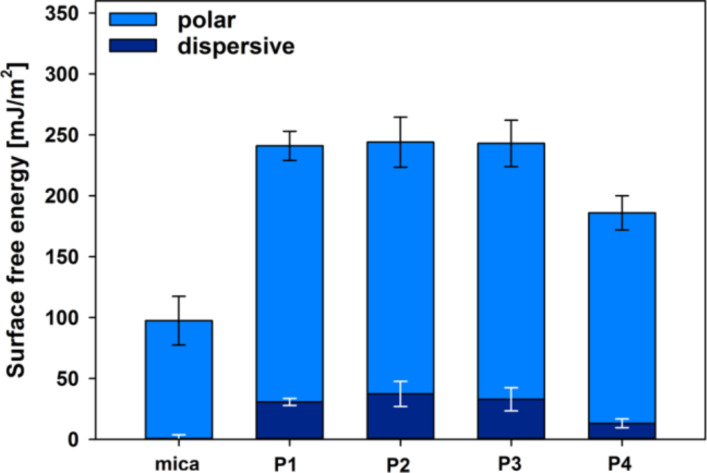
Surface free energy of LPSQ-COOH/X coated on native mica determined by wetting angle measurements.

The surface free energy (γ_S_) of each studied PSAM sample was determined by measuring the contact angle of water and glycerol as reference liquids (sessile drop technique and Owens–Wendt geometric mean Equation S1 described in Supporting Information File 1 [55]). We have previously reported [37–38] that the very good wettability of mica coated with LPSQ-COOH is a result of the structure of adsorbed nanolayers and the fact that carboxylic groups attached to oligomers of LPSQ-COOH adopt a specific conformation at the interface with air. We have analysed the wettability of samples covered with other LPSQ-COOH/X schemes (Figure 2) to find that, in spite of their different morphology, they exhibit almost the same surface energy and the ratio between polar and dispersive forces. The COOH moieties in these polymers bind to the surface of native mica but the remaining polar groups can interact with neighbouring substituents (e.g., dimerization of COOH, or formation of amine salts (–COO^−^NH_2_^+^– and –COO^−^NH_3_^+^–)) and establish a network of hydrogen bonds. The slightly poorer wettability of P4 can be ascribed to the presence of the acetyl group, protecting the NH_2_ function of NAC.

We have analysed the effect of surface roughness on changes in γ_S_ (Figure 1, Figure 2, and Figures S1a, S2a, S3a, S4a and Table S1 in Supporting Information File 1). All surfaces are smooth (with root mean squared roughness factor, *R*_q_ = 0.03 nm for native mica and ≈0.1 ÷ 0.25 nm for PSAMs). No correlation could be found between *R*_q_ and γ_S_. The increase of γ_S_ observed for all studied PSAMs can be tentatively linked to the presence of specific side groups in the adsorbed polymers, capable of effective hydrogen bonding.

### Supramolecular assemblies of LPSQ-COOH/X on primed mica

**Scheme 2:**
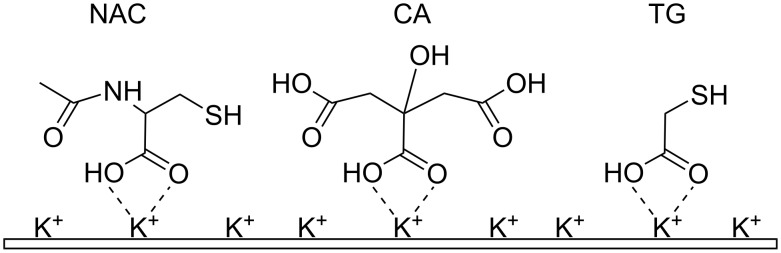
Functionalization of native mica by adsorption of *N*-acetylcysteine (NAC), citric acid (CA) and thioglycolic acid (TG).

The adsorption of LPSQ-COOH/X on native mica is governed by the formation of surface salts–potassium carboxylates. It was of interest to alter these ionic interactions between LPSQ and mica and block the K^+^ ions by adsorption of small molecules [49]. They should be simultaneously capable of the formation of surface salts and hydrogen bonds with functional groups in side chains of LPSQ-COOH/X. Consequently, thioglycolic acid (TG), citric acid (CA) and *N*-acetylcysteine (NAC) were selected and used as primers to modify the surface properties of mica (Scheme 2). They were adsorbed from their diluted solutions in THF or MeOH. The excess of the primer compound was removed by washing the sample with THF.

**Figure 3:**
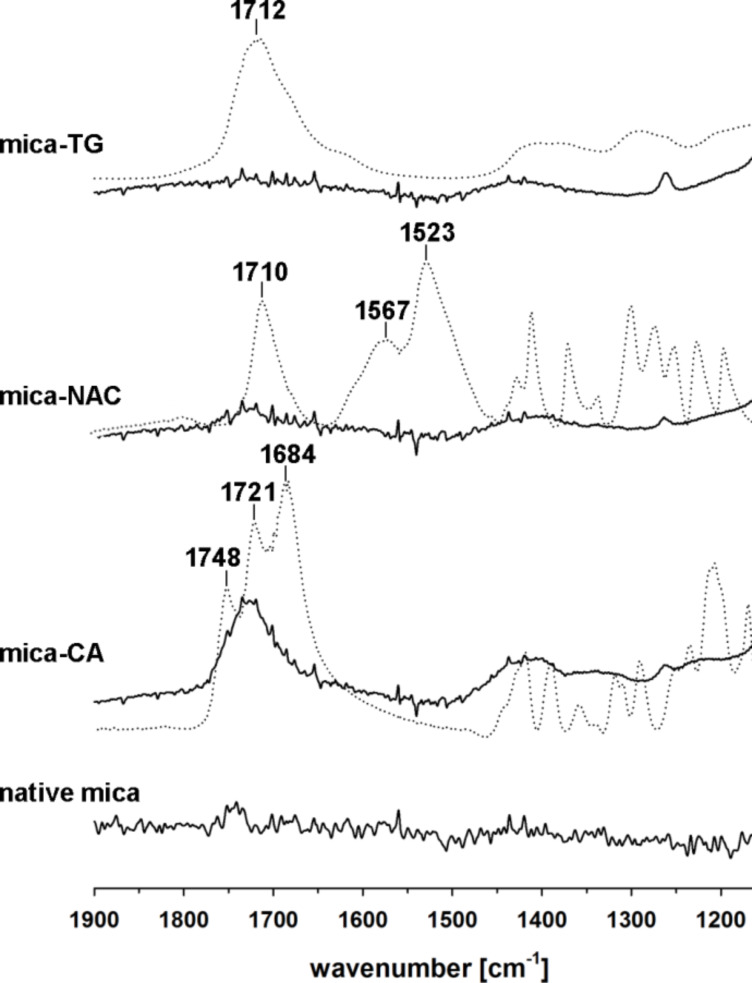
ATR-FTIR spectra (1900–1150 cm^−1^ region) of TG, NAC and CA before (dotted lines) and after their adsorption on muscovite mica.

The formation of surface salts (potassium carboxylates) by the used primers was confirmed by ATR-FTIR analysis (Figure 3). Comparison of the FTIR spectra in the region characteristic to COOH groups shows almost complete disappearance of ν_C=O_ bands observed for the native compounds and formation of diffuse bands characteristic for carboxylates [56]. The only exception is citric acid, which due to the geometric constraints, cannot adsorb on the surface with all of three COOH groups present in the molecule. As expected, part of the COOH groups of adsorbed CA is still available for macromolecules adsorbed as PSAMs. The carboxylic groups of TG are completely transformed into carboxylates. Quite interestingly, two FTIR bands characteristic of the amide bond in NAC (amide I at 1567 cm^−1^ (stretching vibrations of the amide C=O bond) and amide II at 1523 cm^−1^ (bending vibrations of the N–H bond)) disappeared after its adsorption on mica. This phenomenon can be ascribed to changes in geometry of NAC and possible interactions of C=O in the amide unit with K^+^ on the surface of mica.

**Figure 4:**
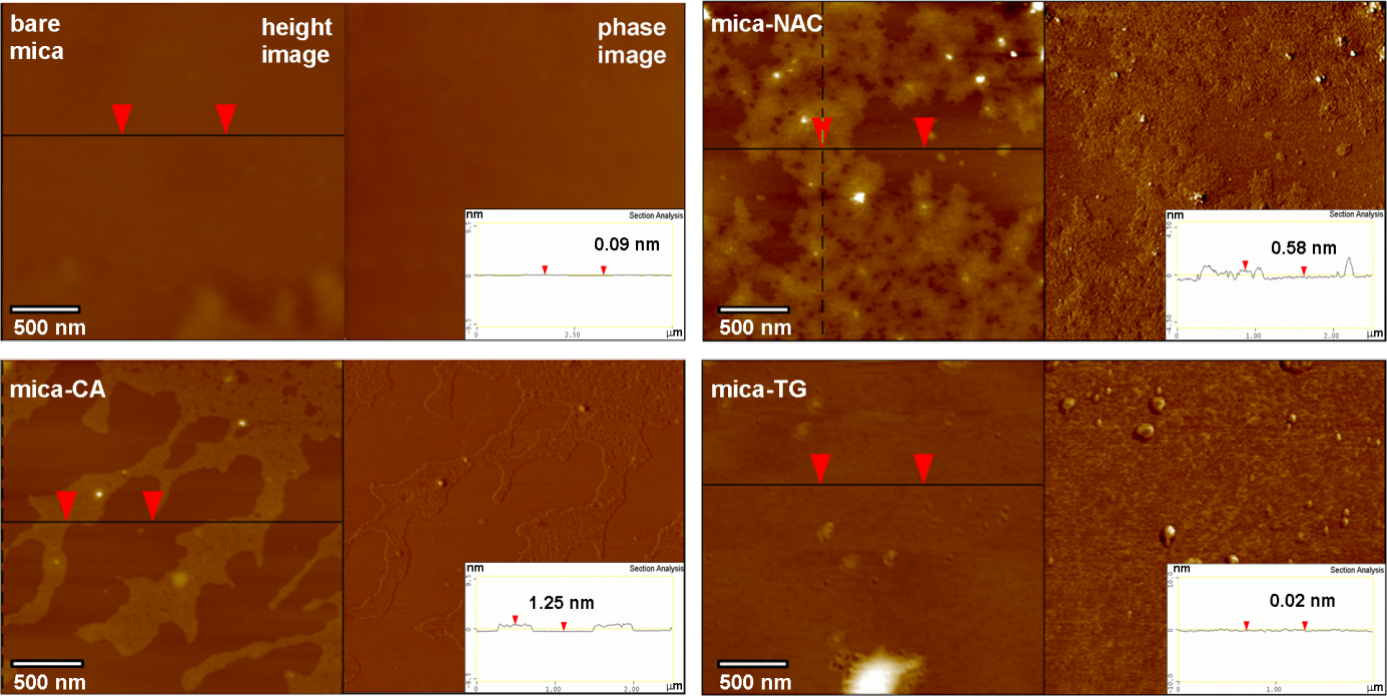
AFM height and phase images of bare mica and mica modified with *N*-acetylcysteine (mica-NAC), citric acid (mica-CA) and thioglycolic acid (mica-TG). The modified samples were prepared by immersion (*t*_i_ = 15 min) of mica in 0.002 M solutions of primers in THF, followed by washing the excess primer by immersion in pure THF (*t*_i_ = 5 s).

AFM was used to analyse the structure of coated samples (Figure 4). We found that the priming compounds evenly cover the surface. The adherence of the used molecules to mica is very high. Dewetting of the adsorbed materials was not observed and the upper layer was not removed or mechanically deformed with the probing tip of the cantilever during the measurement. For NAC and CA, specific structures that suggest formation of multilayered assemblies due to the presence of hydrogen bond accepting groups were observed. The AFM micrographs (Figure 4) show that the top layers have thickness of 0.58 ÷ 1.25 nm, whereas the thickness of a single layer should be close to 0.5 nm (Supporting Information File 1, Figure S6). The *R*_q_ parameter estimated with AFM for these substrates is low (0.24 nm and 0.095 nm for the topmost layers, respectively). For both compounds patches of base layers can be observed that are more smooth (*R*_q_ = 0.077 and 0.047 nm). They do not exhibit clear phase contrast and it cannot be asserted whether they are areas of well-packed molecules or bare mica (*R*_q_ = 0.032 nm for bare mica). The surface of mica covered with TG bearing thiol functions (less effective in hydrogen bonding) is uniform and very smooth (*R*_q_ = 0.117 nm) except for visible drops of excess primer. Priming mica with NAC, CA and TG thus yields smooth, chemo-specific, hydrophilic supports (see later also Figure 9a and Discussion). The abundance of hydroxyl and carboxyl groups on mica treated with CA is responsible for its exceptionally high surface energy.

### AFM studies of LPSQ-COOH/X adsorbed on primed mica

**Figure 5:**
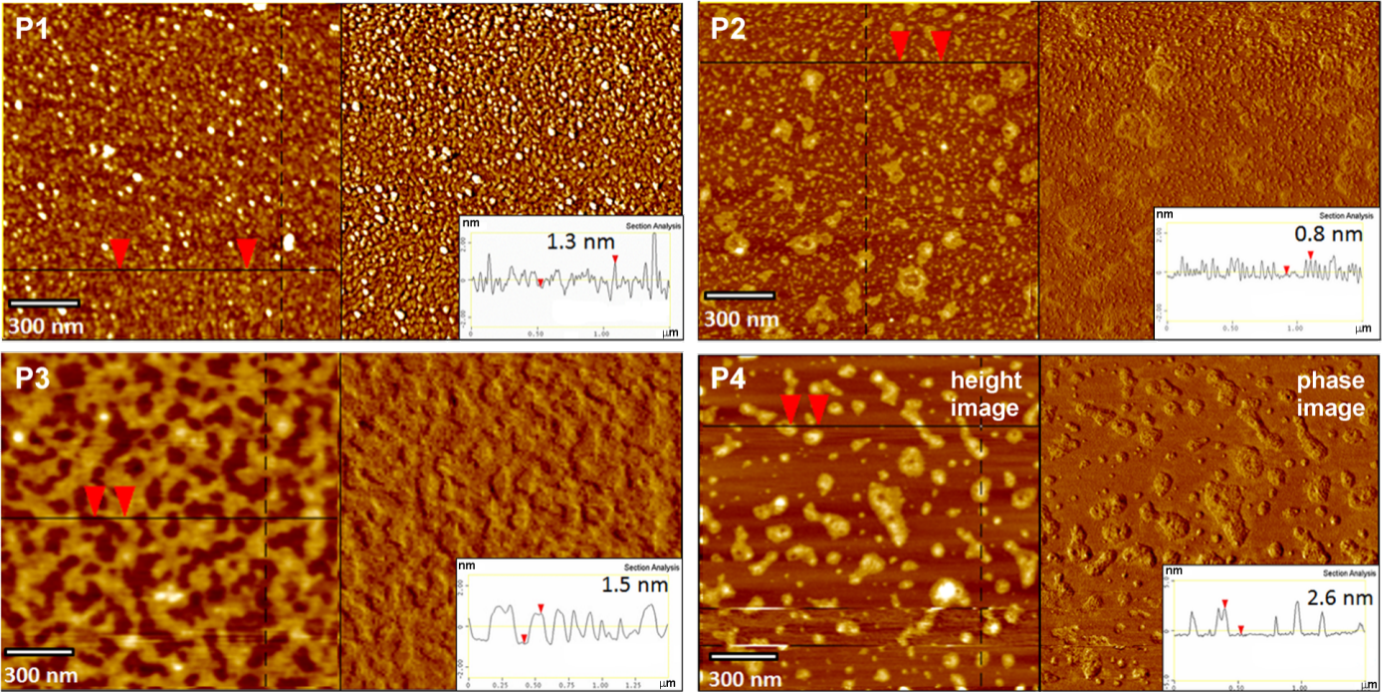
AFM height and phase images and the corresponding surface profiles of P1, P2, P3 and P4, dip-coated on mica modified with citric acid. P1, P3 and P4: 0.045 wt % solutions in THF; P2: 0.045 wt % solution in MeOH; immersion time *t*_i_ = 5 s.

**Figure 6:**
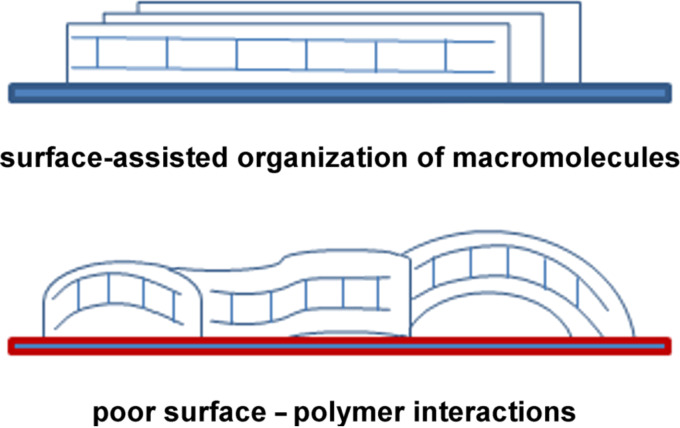
Structure of PSAMs based on LPSQ-COOH/X defined by surface–polymer interactions (composition of side polymer chains and the kind of functional groups on the surface).

After priming, the mica tiles were air-dried and then the polymer layer was adsorbed from diluted solutions of functionalized LPSQ-COOH/X and analysed with AFM and ATR-FTIR. The structure of PSAMs adsorbed on primed mica depends both on the type of primer and the chemical structure of adsorbed macromolecules. The mechanism of adsorption and the character of formed hydrogen bonds is different than that of bare mica. In spite of this, the surface and interlayer adherence is good. The morphology of the samples prepared on muscovite mica treated with citric acid (mica-CA, Figure 5) is governed by the presence of the residual carboxyl groups. The homopolymer P1 (LPSQ-COOH) can form very smooth assemblies on native mica but on mica-CA it tends to coil into fine particles. This can be ascribed to the preferential formation of dimeric hydrogen bonds (intra/intermolecular and surface-P1) involving carboxyl moieties and the lack of predominant, chain-straightening interactions with mica. This phenomenon illustrates the importance of strong surface–adsorbate interactions for the formation of well-assembled PSAMs (Figure 6).

**Figure 7:**
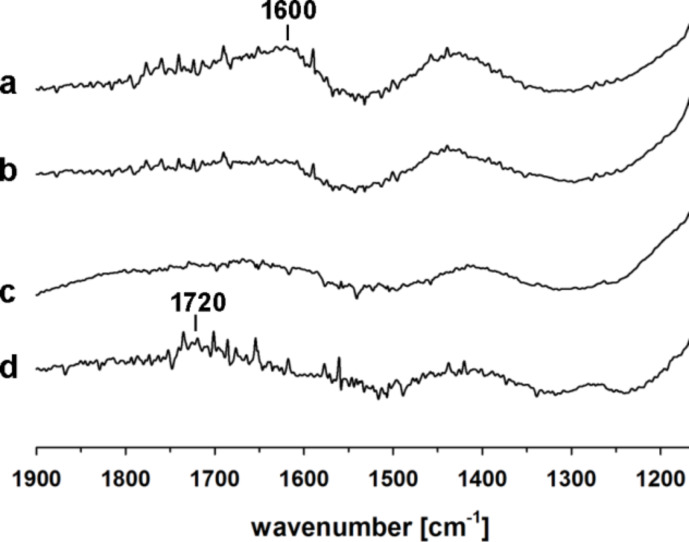
ATR-FTIR spectra (1900–1150 cm^−1^ region) of P1 adsorbed on (a) mica-CA, (b) mica-TG, (c) mica-NAC and (d) native mica.

**Figure 8:**
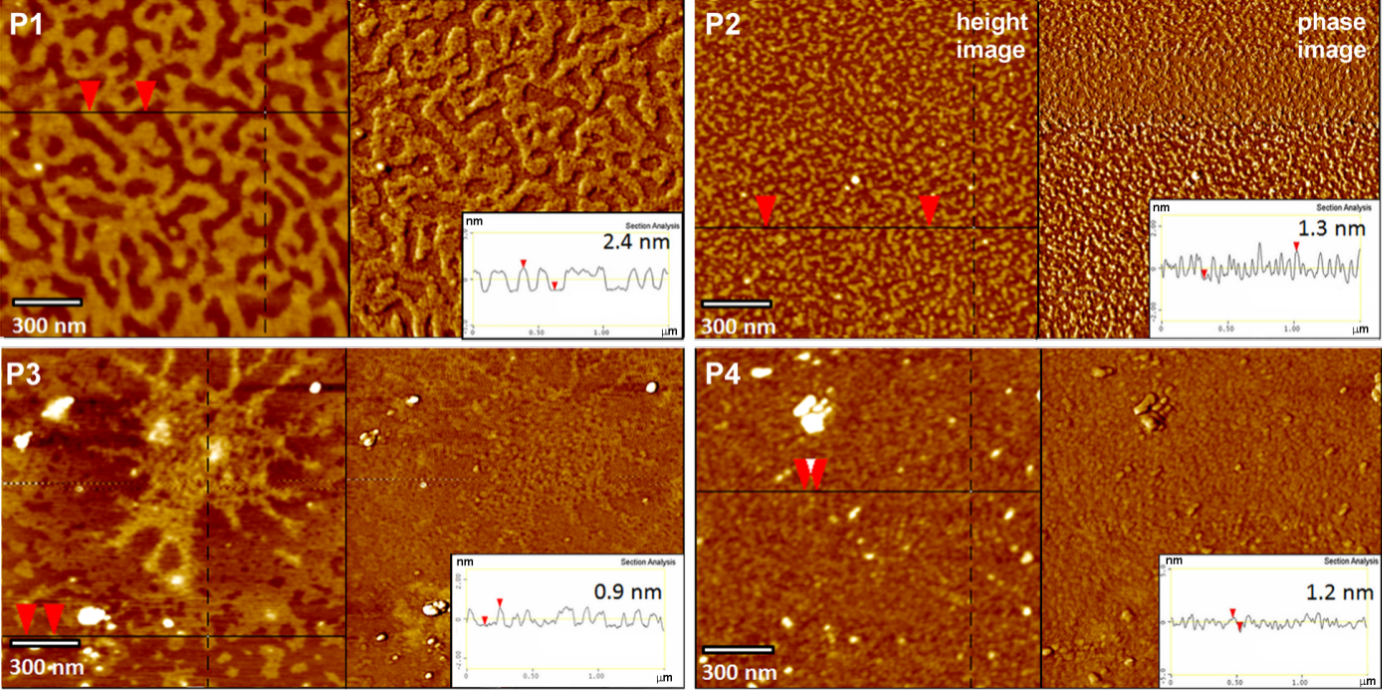
AFM height and phase images and the corresponding surface profiles of P1, P2, P3 and P4, dip-coated on mica modified with thioglycolic acid. P1, P3 and P4: 0.045 wt % solutions in THF; P2: 0.045 wt % solution in MeOH; immersion time *t*_i_ = 5 s.

**Figure 9:**
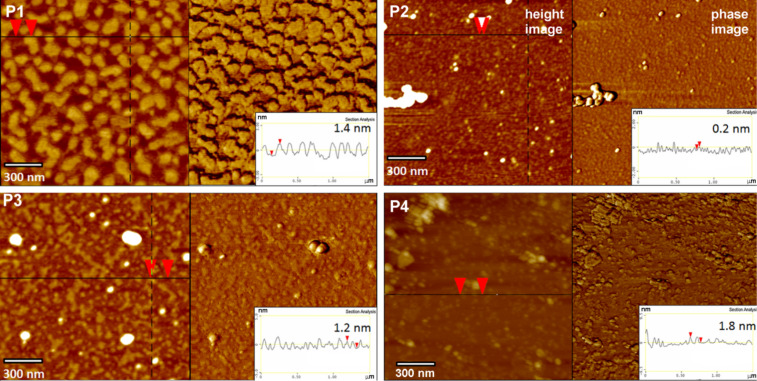
AFM height and phase images and the corresponding surface profiles of P1, P2, P3 and P4, dip-coated on mica modified with *N*-acetylcysteine. P1, P3 and P4: 0.045 wt % solutions in THF; P2: 0.045 wt % solution in MeOH; immersion time *t*_i_ = 5 s.

ATR-FTIR studies in the sensitive region (1900–1150 cm^−1^) indicate a significant alteration in the nature of interactions between the studied macromolecules and the surface (Figure 7 and Supporting Information File 1). The most clear changes can be observed for sample P1 (Figure 7) that binds to the native mica through ionic bonds with the K^+^ layer, and the rest of the COOH groups involved in the intermolecular interactions form a network of hydrogen bonds arranged mostly in linear (catemeric) structures resulting in a characteristic FTIR ν_C=O_ band at ≈1720 cm^−1^ [37]. The thermally induced reshuffling of the catemeric form into an arrangement with dimeric –COOH···HOOC– units results in a shift of the ν_C=O_ band to 1600 cm^−1^ [38]. A similar shift can be found for P1 adsorbed on primed mica. It correlates with the difference in the topographic structure observed by AFM for P1 adsorbed on native and primed mica (Figure 1, Figure 5, Figure 8 and Figure 9). It can be surmised that LPSQ-COOH is anchored on the primed surface (formation of hydrogen bonds with C=O, SH and NH moieties) but instead of producing lamellar structures bound by hydrogen bonds (linear catemeric structures) it adheres to mica as clusters of polymeric chains cross-linked by –COOH···HOOC– dimers or amine salts (–COO^−^NH_2_^+^– and –COO^−^NH_3_^+^–).

Species P2, P3 and P4, having donor/acceptor NH/NH_2_ units, can adsorb on mica-CA via formation of complementary hydrogen bonds with COOH groups, which results in a change of the surface morphology (Figure 5). P3 bearing GSH units forms different structures than P2 and P4, which can be explained by better accessibility of donor/acceptor units in GSH molecules. Cys-HCl and NAC in P2 and P4 are more hindered by the polymer matrix.

A similar trend can be observed for samples adsorbed on supports pretreated with mica-TG or mica-NAC (Figure 8 and Figure 9). Thiol groups are present on both surfaces. Thiols are more nucleophilic than hydroxyls and thus their ability for the formation of hydrogen bonds is different. P1 produces well-ordered layers on mica-TG and multilayered globular formations on mica-NAC. P2 and P4 are well-dispersed on surfaces obtained with those supports. The morphology of surfaces covered with P3 suggests good interaction between GSH units in the polymer and thiol groups on mica.

### Surface energy of LPSQ-COOH/X coated on primed mica

**Figure 10:**
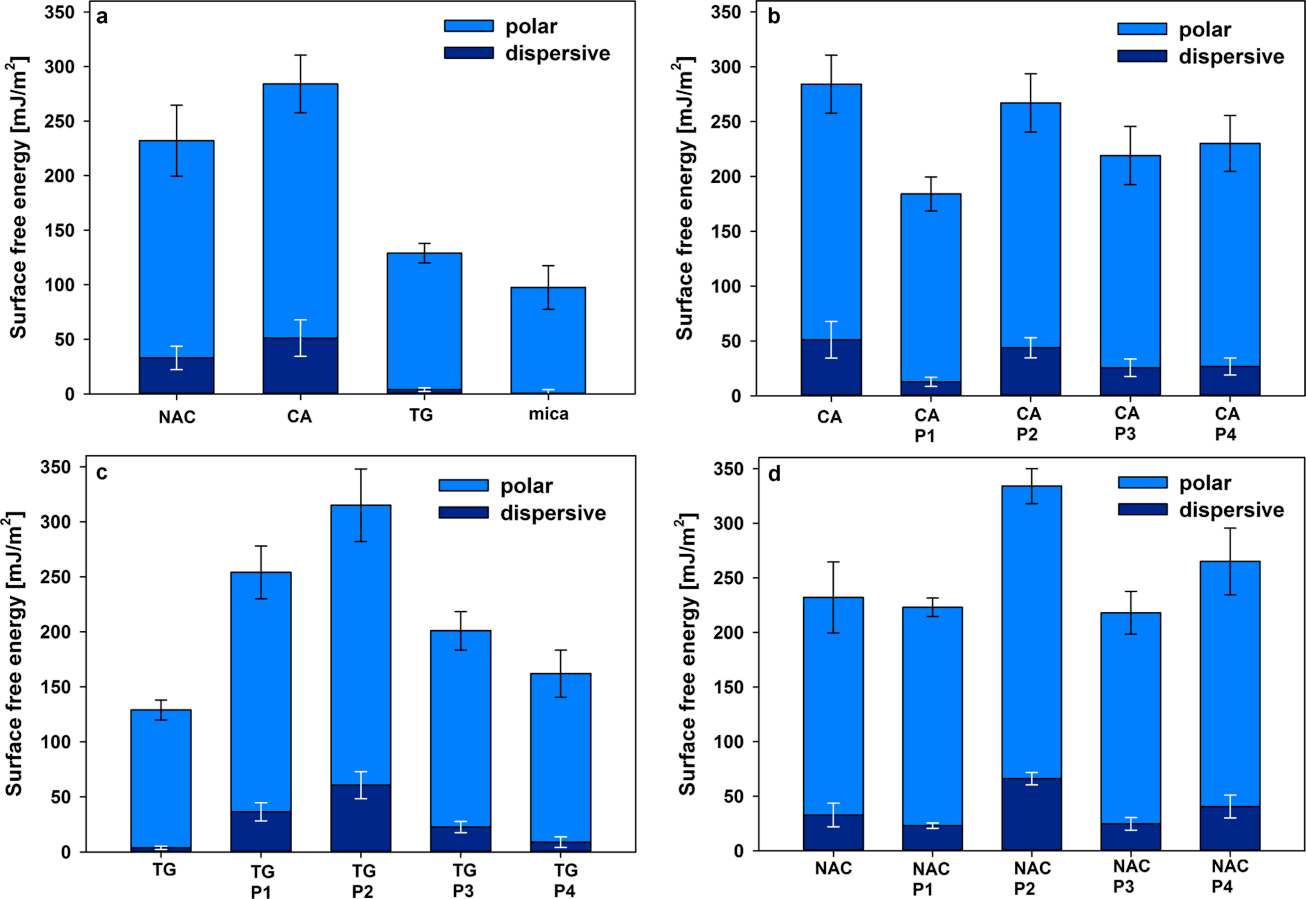
Surface energy of (a) mica pretreated with NAC, CA and TG; (b) P1, P2, P3 and P4 adsorbed on mica-CA; (c) P1, P2, P3 and P4 adsorbed on mica-TG; and (d) P1, P2, P3 and P4 adsorbed on mica-NAC, as determined by wetting angle measurements.

The surface free energy measurements (Figure 10) proved the proposed model of adsorption of NAC, CA and TG (Scheme 2) by ionic interactions of COOH substituents with K^+^ ions on the surface of mica. Such an arrangement of the multifunctional primers exposes reactive polar groups (e.g., SH, NH, OH and COOH). Their ability for the formation of hydrogen bonds with probe liquids (H_2_O and glycerol) defines the wettability and chemical specificity of the modified supports (Figure 10a). The results are in accordance with ATIR-FTIR data (Figure 3). Citric acid, which was shown to adsorb on mica with part of its COOH moieties, gives the most hydrophilic surface.

The analysis of the surface roughness and changes in the free surface energy did not indicate any defined trend that could indicate an effect of surface structure on its wettability. It must be stressed that all the prepared samples exhibit *R*_q_ < 0.5 nm, which is characteristic of smooth surfaces.

The modification of mica-CA by adsorption of LPSQ-COOH/X results in a decrease of the surface free energy (Figure 10b). The most significant effect was observed for P1. It can be explained by the formation of dimeric structures by COOH groups, which was recently reported as the cause for the decrease of surface wettability [38]. The extent of the surface-guided organization of P1 directs its arrangement on the support and changes of the surface energy (Figure 10b,c). P2, in spite of the apparent lack of lamellar organization on the surface, exhibits the highest surface free energy among the studied samples (Figure 10b–d), which can be explained by the presence of the polar amine function. When NH_2_ is protected by an acetyl group (polymer P4), the wettability of the coated samples is lower.

## Conclusion

The structure and properties of PSAMs made of ladder-like oligosilsesquioxanes LPSQ-COOH/X on chemo-reactive supports (bare and functionalized muscovite mica) have been analysed. The AFM studies showed that linear oligomers adsorb on the surface of mica and form various types of structures, depending both on the morphology of LPSQ-COOH/X and the chemical specificity of the support. The functional groups in side chains have a significant impact on the arrangement of macromolecules, surface pattern and hydrophilicity. The distribution of the studied macromolecules within the adsorbed PSAMs is a consequence of both polymer–substrate as well as inter- and intramolecular bonding. The homopolymer LPSQ-COOH can form the smoothest layers with macromolecules arranged horizontally in the monolayer due to the specific mechanism of their adsorption on mica. An alternative adsorption mechanism and the shifting of the orientation of the silsesquioxane chains towards more mushroom-like shapes allows for the possibly for other LPSQ-COOH/X schemes. It was also found that priming the substrate with small organic compounds can alter the structure of the adsorbed polymeric films due to the change of specific interactions between the polymer and the surface. The presented, simple approach for the preparation of hydrophilic, nanopatterned surfaces rich in organic polar groups can be especially useful in bioengineering.

## Supporting Information

The supporting information features all experimental procedures, characterization methods, and NMR spectral data for the prepared LPSQ-COOH/X materials in addition to the ATR-FTIR spectra of P1, P2, P3 and P4, adsorbed on mica-CA, mica-TG and mica-NAC.

File 1 Experimental part.
