# Supplementary material for: Nanostructured surfaces by supramolecular self-assembly of linear oligosilsesquioxanes with biocompatible side groups
Source: Beilstein J Nanotechnol. 2015 Dec 11;6:2377–87. doi: 10.3762/bjnano.6.244 (PMC4685769; doi:10.3762/bjnano.6.244)
Supplement: File 1 — Experimental part. [file Beilstein_J_Nanotechnol-06-2377-s001.pdf]

Supporting Information

for

Nanostructured surfaces by supramolecular self-assembly of linear oligosilsesquioxanes with biocompatible side groups

Maria Nowacka, Anna Kowalewska*, and Tomasz Makowski

Address: Centre of Molecular and Macromolecular Studies, Polish Academy of Sciences, Sienkiewicza 112, 90-363 Łódź, Poland

Email: Anna Kowalewska - anko@cbmm.lodz.pl

Experimental part

General information

Materials

Triethoxyvinylsilane (Aldrich, 97%), hexamethyldisilazane (Fluka Analytical, ≥98%, GC), potassium hydroxide (Aldrich, ACS reagent, ≥85, pellets), acetic acid (Aldrich, ≥99%, ReagentPlus®), magnesium sulphate (VI) $MgSO_4$ (Chempur, pure)] DMPA (Acros Organics, 99%), thioglycolic acid (TG, Fisher Scientific, pure p.a.) *N*-acetylcysteine (NAC, Aldrich, Sigma grade, >99%), L-glutathione reduced (GSH, Aldrich), L-cysteine hydrochloride (Cys-HCl, Aldrich, anhydrous, ≥98%) and citric acid monohydrate (CA, Chempur, pure p.a.), were used as received.

Solvents [ethyl acetate (Chempur, pure p.a.), hexane (Aldrich, Chromasolv® for HPLC, ≥97%, GC), n-pentane (Chempur, pure p.a), tetrahydrofuran (THF) (Chempur, pure p.a.), dichloromethane (Chempur, pure p.a.), methanol (Chempur, pure p.a.)], dimethylformamide (DMF) (POCH, pure p.a.) were purified according to the literature procedures [Armarego, W. L. F.; Chai Ch. L. L. "Purification of laboratory chemicals", 5th edition, Elsevier Science, **2003**].

Muscovite mica tiles (V-1 grade, SPI Supplies/Structure Probe, Inc.) were freshly cleaved before use by removing the top layer with an adhesive tape.

Analytical methods

Liquid state NMR (^{1}H, ^{13}C and ^{29}Si NMR) spectra for the precursors and condensed soluble materials were recorded in $CDCl_3$ or THF-d_8 as a solvent on a Bruker DRX-500 MHz spectrometer, with TMS as the reference. Solid-state ^{13}C and ^{29}Si CPMAS NMR spectra were recorded with high power decoupling (HP Dec) mode on an AV-400 Bruker spectrometer at 59.627 MHz. The peak positions were referenced to the signal of Q_8M_8 (trimethylsilyl ester of cubic octameric silicate) as standard.

Mass spectrometry measurements (MALDI-TOF) were performed for LPSQ-Vi using a Voyager-Elite (PerSeptive Biosystems, USA) time-of-flight instrument equipped with a pulsed N_2 laser (337 nm, 4 ns pulse width) and time-delayed extraction ion source. An accelerating voltage of 20 kV was applied. Mass spectra were recorded in the linear positive ion mode using 1,8-dihydroxy-9-anthracenone (ditranol, DT) as the matrix and LiCl as the cationization agent.

Size exclusion chromatography (SEC) was performed for LPSQ-Vi using Agilent 1100 series chromatograph composed of degasser, pump, and autosampler. Two PL Gel 5 µm MIXED-C columns (7.8 x 300 mm) were used in a series (27 °C). RI (OPTILAB T-rex, Wyatt) and MALLS (DAWN HEOS, II, Wyatt, λ = 682 nm) were used as detectors. Dichloromethane was used as mobile phase at the flow rate of 0.8 mL/min. Molecular masses were derived from a

calibration curve based on polystyrene standards. Phase transitions of polymers were studied by differential scanning calorimetry (DSC) technique (DuPont 2000 thermal analysis system).

Fourier transform infrared spectra were collected on a Nicolet 6700 spectrometer. The technique of attenuated total reflectance (ATR) was applied for IR measurements, using germanium crystal attachment together with the mercury cadmium telluride detector (MCTD). The spectra were obtained by adding 64 scans at a resolution of 2 cm^{-1}.

Surface characterization

Atomic Force Microscopy (AFM) images were recorded under ambient atmosphere using Nanoscope IIIa, MultiMode microscope (Veeco, Santa Barbara, CA). The analysis (tapping mode) was carried out at room temperature (unless otherwise indicated). The probes were commercially available rectangular silicon cantilevers (RTESP from Veeco) with nominal radius of curvature in the 10 nm range, spring constant 20–80 N m^{-1}, and a resonance frequency lying in the 264–369 kHz. The images were recorded with the highest available sampling resolution, that is, 512 x 512 data points. The applied eight ranges are −5 nm ÷ 5 nm, the offset of phase images is 15°.

Root mean square roughness (RMS = R_q) was used as a statistical measure for estimation of the surface smoothness [De Oliveira R.R.L., Albuquerque D.A.C., Cruz T.G.S., Yamaji F.M., Leite F.L., Chapter 7 “Measurement of the Nanoscale Roughness by Atomic Force Microscopy: Basic Principles and Applications” in Atomic Force Microscopy - Imaging, Measuring and Manipulating Surfaces at the Atomic Scale, Ed. Bellitto V. InTech, **2012**].

R_q can be defined as the standard deviation of the Z-values within the box cursor and is calculated using Equation S1 where Z_i is the current Z-value, and N is the number of points within the box cursor. R_q is very sensitive to height deviations (peaks and valleys) in surface roughness profile due to the squaring of the values of amplitude in its calculation. The scan size of the analysed areas can be found in Table S1 and Figures S1–5 (the baseline was set automatically for the height level 0).

$$R_q = \sqrt{\frac{\sum (Z_i)^2}{N}} \quad \text{(S1).}$$

Contact-angle measurements: static contact angles of sessile droplets of deionized water and glycerol (Chempur, pure p.a., anhydrous) at the film–air interface were measured at room temperature with a Rame-Hart NRL contact-angle goniometer equipped with a video camera JVC KYF 70B and drop-shape analysis software. The measurements were performed on untreated bare mica, mica coated with small organic primers, native and primed mica coated with PSAMs. Static, advancing, and receding contact angles were measured right after the deposition of the liquid onto the film surface. The values of contact angles (standard deviation estimated) are an average of at least three measurements taken on different areas of the same sample. They are reported for a short contact time, strictly

observed to diminish the effect of surface accommodation. Surface energies were estimated by the Owens–Wendt method using the values of advancing angles [Owens, D. K.; Wendt, R. C. *J. Appl. Polym. Sci.*, **1969**, *13*, 1741–1747]. The Owens–Wendt equation, Equation S2, where where θ is the measured contact angle of a liquid on the surface, and γ_L is the surface tension of the liquid, allows for division of the surface free energy (γ_S) into the dispersive ($\gamma^D{}_S$) and polar ($\gamma^P{}_S$) components ($\gamma_S = \gamma^D{}_S + \gamma^P{}_S$).

$$(1+\cos\theta)\gamma_L = 2\sqrt{\gamma_S^D\gamma_L^D} + 2\sqrt{\gamma_S^P\gamma_L^P} \qquad \text{(S2)}$$

Polar and dispersive components were separated automatically by the programme "Drop" (version 2.1, written and authored by Dr. hab. Stanisław Sosnowski at Centre of Molecular and Macromolecular Studies, PAS) that was used for calculations of γ_S.

Experimental procedures

Synthesis of oligo(vinylsilsesquioxanes) (LPSQ-Vi): the oligomers were prepared according to the previously described procedure [Kowalewska, A.; Nowacka, M. *Silicon*, **2015**, *7*, 133–146]. The obtained product is a viscous, colourless liquid (3.3 g, Y = 67.5%).

^{1}H NMR ($CDCl_3$), δ [ppm]: 0.09 (s. $OSiMe_3$); 5.19 (m. $-CH=CH_2$)

^{13}C NMR ($CDCl_3$), δ [ppm]: 1.6 ($OSiMe_3$); 130.2 (=CH-);135.9 ($=CH_2$)

^{29}Si NMR ($CDCl_3$); δ [ppm]: –81.3 ($H_2C=CH-SiO_{3/2}$); 10.6 ($OSiMe_3$)

SEC in CH_2Cl_2 (RI): Mn= 1000, Mw= 1200, PDI=1.2, (MALLS): Mn=2000, Mw=3200, PDI=1.6.

MALDI-TOF mass spectrometry was used as a method for the determination of the structure of LPSQ-Vi. Linear oligomers with chain ends terminated with trimethylsilyl groups were detected.

Synthesis of oligo[2-(carboxymethylthio)ethylsilsesquioxanes] (P1)*:* the oligomers were prepared according to the previously described procedure [Kowalewska, A.; Nowacka, M.; Tracz A.; Makowski, T. *Soft Matter*, **2015**, *11*, 4818-4829] by UV initiated thiol-ene addition of thioglycolic acid to LPSQ-Vi. DMPA (0.25 mmol) was added to a solution of thioglycolic acid (17.6 mmol) and LPSQ-Vi (1g, 12.6 mmol Vi groups) in dry THF (15 mL), placed in a quartz vessel. The mixture was irradiated for 30 min with UV light (350 nm). Volatiles were then removed under reduced pressure and the residue was dissolved in THF (2 mL) and precipitated into n-pentane (100 mL). The purification procedure was repeated thrice. The precipitate was dried under high vacuum at room temperature to a constant weight. A viscous polymer was obtained (1.8 g, Y = 83%).

^{1}H NMR (THF-d8), δ [ppm]: 0.17 (s. $OSiMe_3$); 1.08 (m. $SiCH_2$); 2.79 (CH_2S); 3.20 (m. SCH_2); 8.30 (COOH)

^{13}C NMR (CD_3OD), δ [ppm]: -0.5 ($OSiMe_3$); 11.4 ($SiCH_2$); 25.3 (CH_2S); 31.6 (SCH_2); 170.9 (C=O)

^{29}Si NMR (CD_3OD); δ [ppm]: –70 (-CH_2-$SiO_{3/2}$); 11 ($OSiMe_3$)

Synthesis of copolymeric derivatives of cysteine hydrochloride and thioglycolic acid (P2)*:* the oligomers were prepared by UV initiated thiol-ene addition of cysteine hydrochloride followed by addition of thioglycolic acid to LPSQ-Vi. DMPA (0.031 mmol) was added to a solution of cysteine hydrochloride (0.38 mmol) and LPSQ-Vi (0.15 g, 1.9 mmol Vi groups) in dry DMF (19 mL), placed in a quartz vessel. The mixture was irradiated for 45 min with UV light (350 nm). Volatiles were then removed under reduced pressure and the residue was dissolved in MeOH (18 mL) and placed in another quartz vessel. Thioglycolic acid (0.14 g, 1.52 mmol) and DMPA (10 mg) were added and the mixture was irradiated for 30 min with UV light. Volatiles were then removed under reduced pressure and the residue was dissolved in MeOH and precipitated into hexanes. The purification procedure was repeated thrice. The precipitate was dried under high vacuum at room temperature to a constant weight. A solid polymer was obtained (0.34 g, Y = 97%).

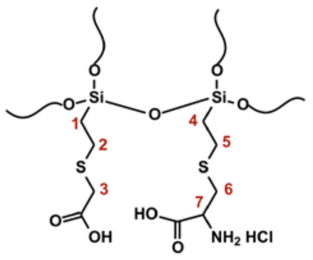

^{1}H NMR (CD_3OD), δ [ppm]: 0.15 (s. $OSiMe_3$); 1.0 [m. $SiCH_2$(Cys-HCl) *4*]; 1.10 [m. $SiCH_2$(TG) *1*]; 2.50-2.80 [m. CH_2S(TG/ Cys-HCl) *2+5*]; 3.20-3.40 [m. SCH_2(TG/Cys-HCl) *3+6*]; 4.2 [m. CH(Cys-HCl) 7]; 8.2-8.5 (COOH)

^{13}C HP Dec, δ [ppm]: -0.5 ($OSiMe_3$); 11.0 CH_2Si(TG/Cys-HCl) *1+4*; 25.0 CH_2S(TG/Cys-HCl) *2+5*; 28.0 CH_2S(Cys-HCl) *6*; 32.0 SCH_2(TG) *3*; 60.0 CH(Cys-HCl) *7*; 169.1, 172.5 (C=O)

^{29}Si HP Dec; δ [ppm]: -70 (-CH_2-$SiO_{3/2}$); 10 ($OSiMe_3$)

Synthesis of copolymeric derivatives of glutathione and thioglycolic acid (P3)*:* the oligomers were prepared by UV initiated thiol-ene addition of glutathione followed by addition of thioglycolic acid to LPSQ-Vi. DMPA (0.11 mmol) was added to a solution of glutathione (0.19 mmol) and LPSQ-Vi (75 mg, 0.95 mmol Vi groups) in dry DMF (32 mL), placed in a quartz vessel. The mixture was irradiated for 60 min with UV light (350 nm). Volatiles were then removed under reduced pressure and the residue was dissolved in THF (30 mL) and placed in another quartz vessel. Thioglycolic acid (0.07 g, 0.76 mmol) and DMPA (28 mg) were added and the mixture was irradiated for 45 min with UV light. Volatiles were then removed under reduced pressure and the residue was dissolved in THF and precipitated into hexanes. The purification procedure was repeated thrice. The precipitate was dried under high vacuum at room temperature to a constant weight. A solid polymer was obtained (0.20 g, Y = 98%).

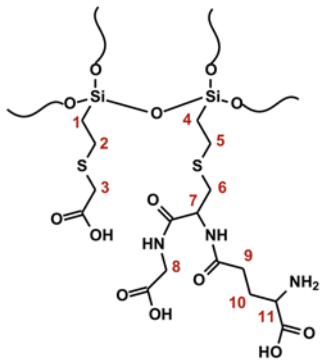

^{1}H NMR (THF-d8), δ [ppm]: 0.19 (s. $OSiMe_3$); 0.9 [m. $SiCH_2$(GSH) *4*]; 1.10 [m. $SiCH_2$(TG) *1*]; 2.8 [m. CH_2S(TG)/SCH_2(GSH) *2+5+6*]; 2.8-3.1 [m. CH_2(GSH)/CH_2(GSH) *9+10*]; 3.2 [s. SCH_2(TG) *3*]; 3.8-4.0 [m. CH(GSH)/CH(GSH)/CH_2(GSH) *7+8+11*]; 7.2-8.2 (COOH)

[13]C HP Dec, δ [ppm]: -0.5 ($OSiMe_3$); 11.0 [CH_2Si(TG/GSH) *1+4*]; 26.0 [CH_2S(TG)/CH_2S(GSH)/CH_2(GSH)] *2+5+10*]; 32.0 [SCH_2(TG/GSH)/CH_2(GSH) *3+6+9*]; 49.2 CH_2(GSH) *8*; 53.5 [CH(GSH)/CH(GSH) *7+11*]; 168.0, 170.6, 171.8, 175.8 (C=O)

[29]Si HP Dec; δ [ppm]: -71 ($-CH_2-SiO_{3/2}$); 10 ($OSiMe_3$)

Synthesis of copolymeric derivatives of N-acetylcysteine and thioglycolic acid (P4)*:* the oligomers were prepared by UV initiated thiol-ene addition of *N*-acetylcysteine followed by addition of thioglycolic acid to LPSQ-Vi. DMPA (0.098 mmol) was added to a solution of *N*-acetylcysteine (0.50 mmol) and LPSQ-Vi (0.20 g, 2.52 mmol Vi groups) in dry THF (35 mL), placed in a quartz vessel. The mixture was irradiated for 60 min with UV light (350 nm). Thioglycolic acid (0.19 g, 2.02 mmol) and DMPA (25 mg) were added and the mixture was irradiated for 45 min with UV light. Volatiles were then removed under reduced pressure and the residue was dissolved in THF and precipitated into hexanes. The purification procedure was repeated thrice. The precipitate was dried under high vacuum at room temperature to a constant weight. A solid polymer was obtained (0.33 g, Y = 70%).

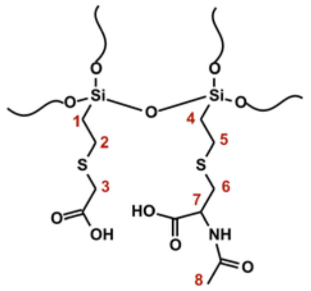

^{1}H NMR (THF-d8), δ [ppm]: 0.14 (s. $OSiMe_3$); 0.9 [m. $SiCH_2$(NAC) *4*]; 1.10 [m. $SiCH_2$(TG) *1*]; 2.10 [s. CH_3(NAC) *8*]; 2.60-2.80 [m. CH_2S(TG/NAC) *2+5*]; 3.20-3.45 [m. SCH_2(TG/NAC) *3+6*]; 4.7 [m. CH(NAC) *7*]; 8.0-8.5 (COOH)

[13]C HP Dec, δ [ppm]: -0.4 ($OSiMe_3$); 9.0 [CH_2Si(TG/NAC) *1+4*]; 23.0 CH_3(NAC) *8*; 26.0 [CH_2S(TG/NAC) *2+5*]; 28.0 SCH_2 (NAC) *6*; 32.0 SCH_2(TG) *3*; 66.0 CH(NAC) *7*; 168.0, 170.8, 172.1 (C=O)

[29]Si HP Dec; δ [ppm]: -70 ($-CH_2-SiO_{3/2}$); 11 ($OSiMe_3$)

Coating native mica with small organic compounds: NAC, TG or CA were dissolved in dry THF at 0.04 wt % concentration. The solutions were filtered through a 0.2 mm PTFE filter and placed in a vessel. Dip coating was carried out at room temperature by the immersion of a freshly cleaved muscovite mica support in the polymer solution for 15 min. The excess of primer compound on a mica surface was removed by washing samples in pure THF for 5 s. The samples were dried under ambient conditions for 24 hours before being used as supports for PSAMs.

Preparation of thin polysilsesquioxane films on solid supports: polymers (P1, P2, P3, P4) were dissolved in dry solvents (P1, P3, P4 in THF, P2 in methanol) at 0.04 wt % concentration. The solutions were filtered through 0.2 mm PTFE filters and placed in separate vessels. Dip coating was carried out at room temperature by the immersion of a freshly cleaved or primed muscovite mica support in the polymer solution for 5 s. All supports were mounted and moved vertically with a motorized linear slide (Zaber Technologies Inc.) (rate of immersion/extraction = 4 mm/s). The volume of liquid and the immersion level were constant. Dip-coated supports were then placed in a closed container and left for drying at room temperature for one day. Their surface was analyzed with AFM (tapping mode). Surface energy of all samples was estimated by contact angle measurements using water and glycerol as probe liquids.

Table S1: Root mean squared surface roughness (R_q) of the studied samples.

Sample	Image R_q [nm]	ΣIPSA [µm^2]	R_q in region (1) [nm]	SPSA (1) [µm^2]	R_q in region (2) [nm]	SPSA (2) [µm^2]
bare mica	0.107	0.701 / 1.69	0.032	0.044 / 0.199	-	-
mica-NAC	0.301	3.150 / 6.25	0.077	0.067 / 0.067	0.240	0.169 / 0.319
mica-CA	0.191	3.580 / 6.25	0.047	0.082 / 0.115	0.095	0.046 / 0.072
mica-TG	0.169	2.930 / 4.00	0.117	0.206 / 0.281	-	-
mica-P1	0.236	1.849 / 4.00	0.147	0.006 / 0.229	-	-
mica-P2	0.142	1.270 / 2.25	0.104	0.118 / 0.228	-	-
mica-P3	0.266	1.410 / 2.25	0.258	0.055 / 0.225	-	-
mica-P4	0.255	1.230 / 2.25	0.208	0.115 / 0.225	-	-
mica-NAC-P1	0.302	1.100 / 2.25	0.335	0.133 / 0.275	-	-
mica-NAC-P2	0.113	0.993 / 2.25	0.089	0.133 / 0.269	-	-
mica-NAC-P3	0.264	1.390 / 2.25	0.244	0.116 / 0.187	-	-
mica-NAC-P4	0.792	1.250 / 2.25	0.300	0.151 / 0.246	-	-
mica-CA-P1	0.210	1.100 / 2.25	0.213	0.098 / 0.207	-	-
mica-CA-P2	0.082	0.403 / 2.25	0.067	0.047 / 0.257	-	-
mica-CA-P3	0.278	10.60 / 25.0	0.269	0.410 / 0.187	-	-
mica-CA-P4	0.292	1.440 / 2.25	0.203	0.169 / 0.251	-	-
mica-TG-P1	0.382	0.972 / 2.25	0.369	0.112 / 0.257	-	-
mica-TG-P2	0.149	1.070 / 2.25	0.146	0.118 / 0.235	-	-
mica-TG-P3	0.186	1.130 / 2.25	0.193	0.006 / 0.056	0.135	0.123 / 0.236
mica-TG-P4	0.233	1.240 / 2.25	0.226	0.147 / 0.246	-	-

ΣIPSA - total image surface area (projected / complete selected area)

SPSA - selected surface area (projected / complete selected area)

R_q - root mean square roughness (projected area)

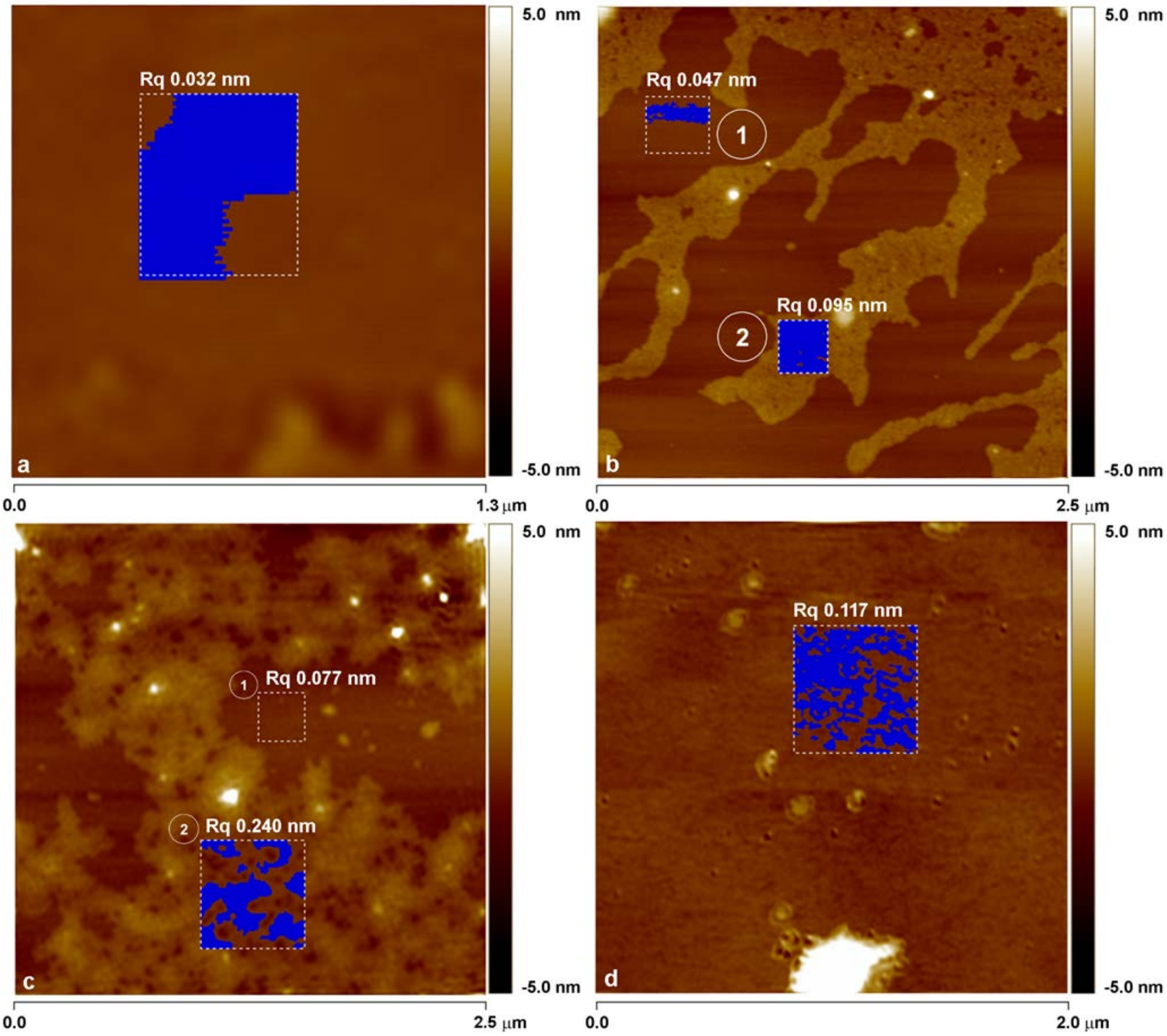

Figure S1: Analysis of surface root mean square roughness (R_q) for bare mica (a) and primed supports: (b) mica-CA, (c) mica-NAC, (d) mica-TG.

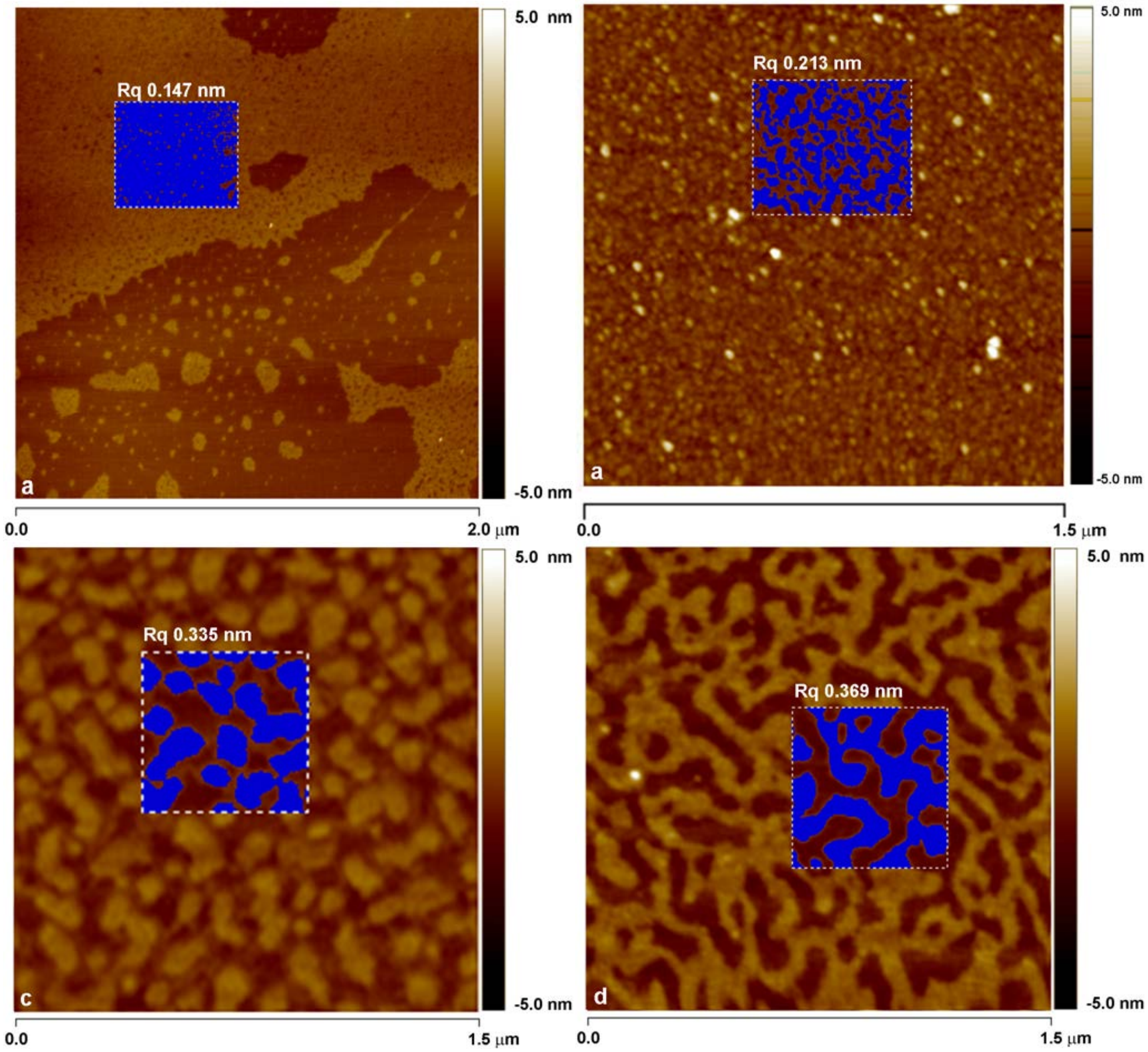

Figure S2: Analysis of surface root mean square roughness (R_q) for PSAMs of P1 coated on bare mica (a) and supports primed with (b) CA (c) NAC (d) TG.

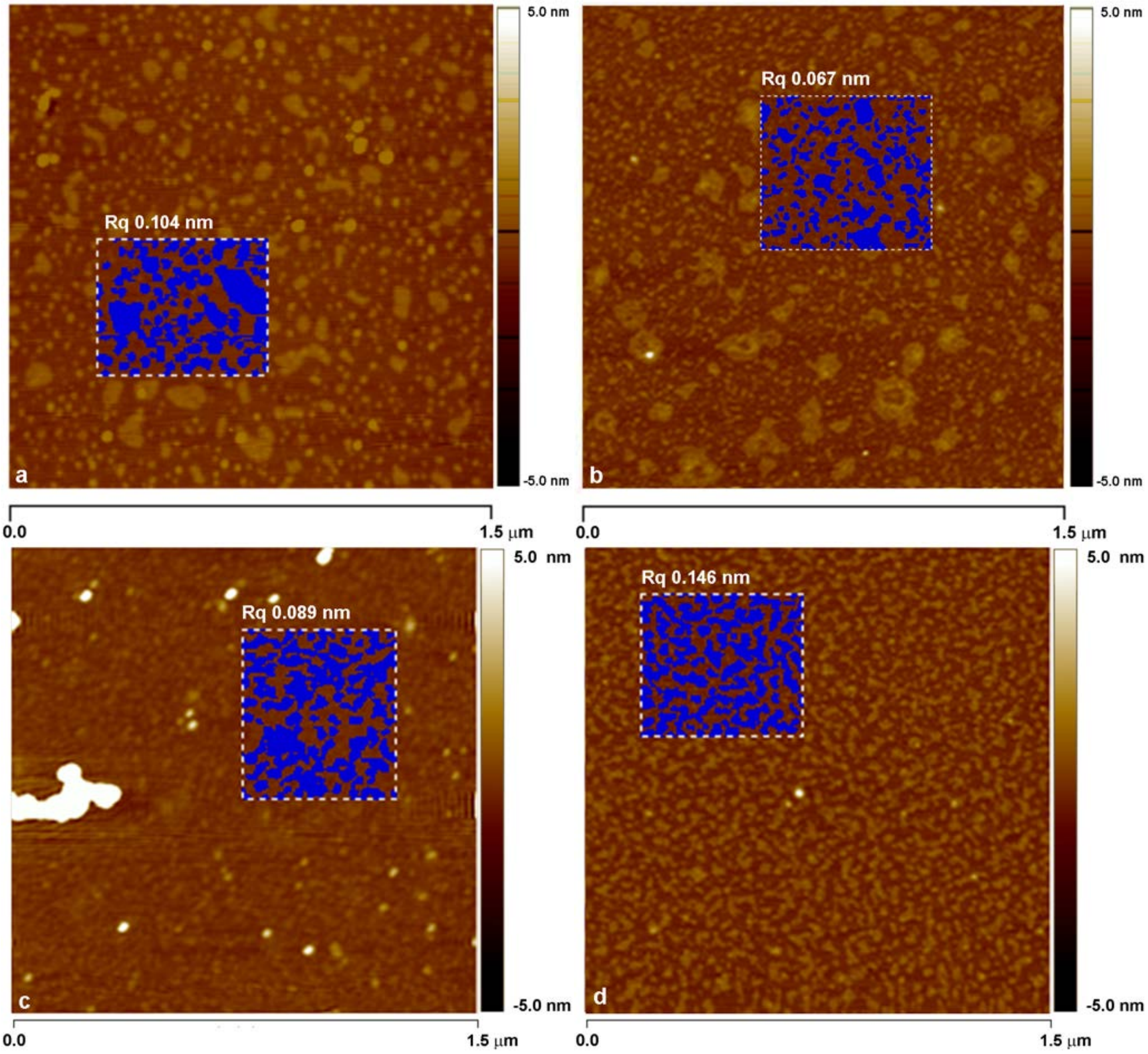

Figure S3: Analysis of surface root mean square roughness (R_q) for PSAMs of P2 coated on bare mica (a) and supports primed with (b) CA (c) NAC (d) TG.

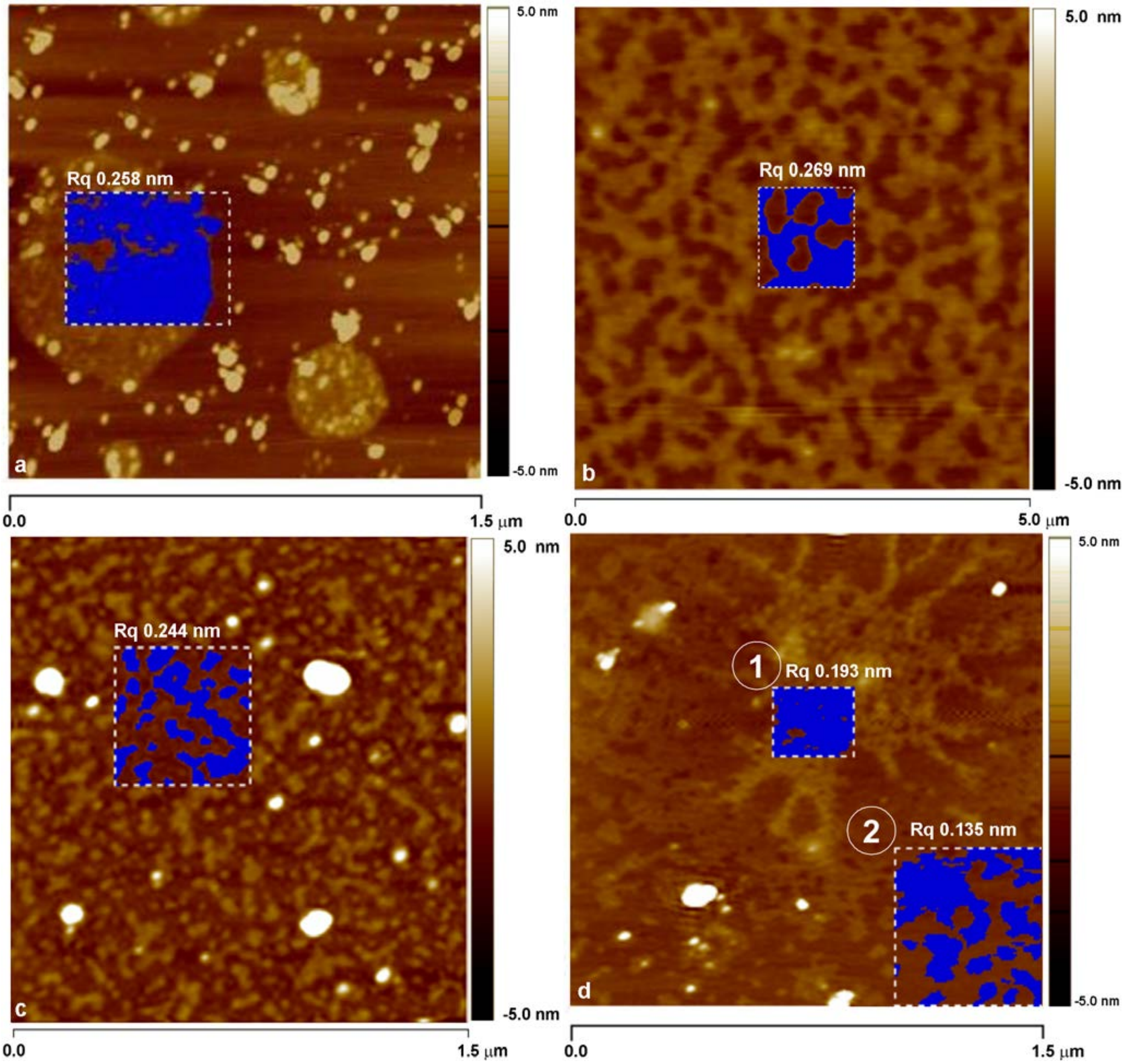

Figure S4: Analysis of surface root mean square roughness (R_q) for PSAMs of P3 coated on bare mica (a) and supports primed with (b) CA (c) NAC (d) TG.

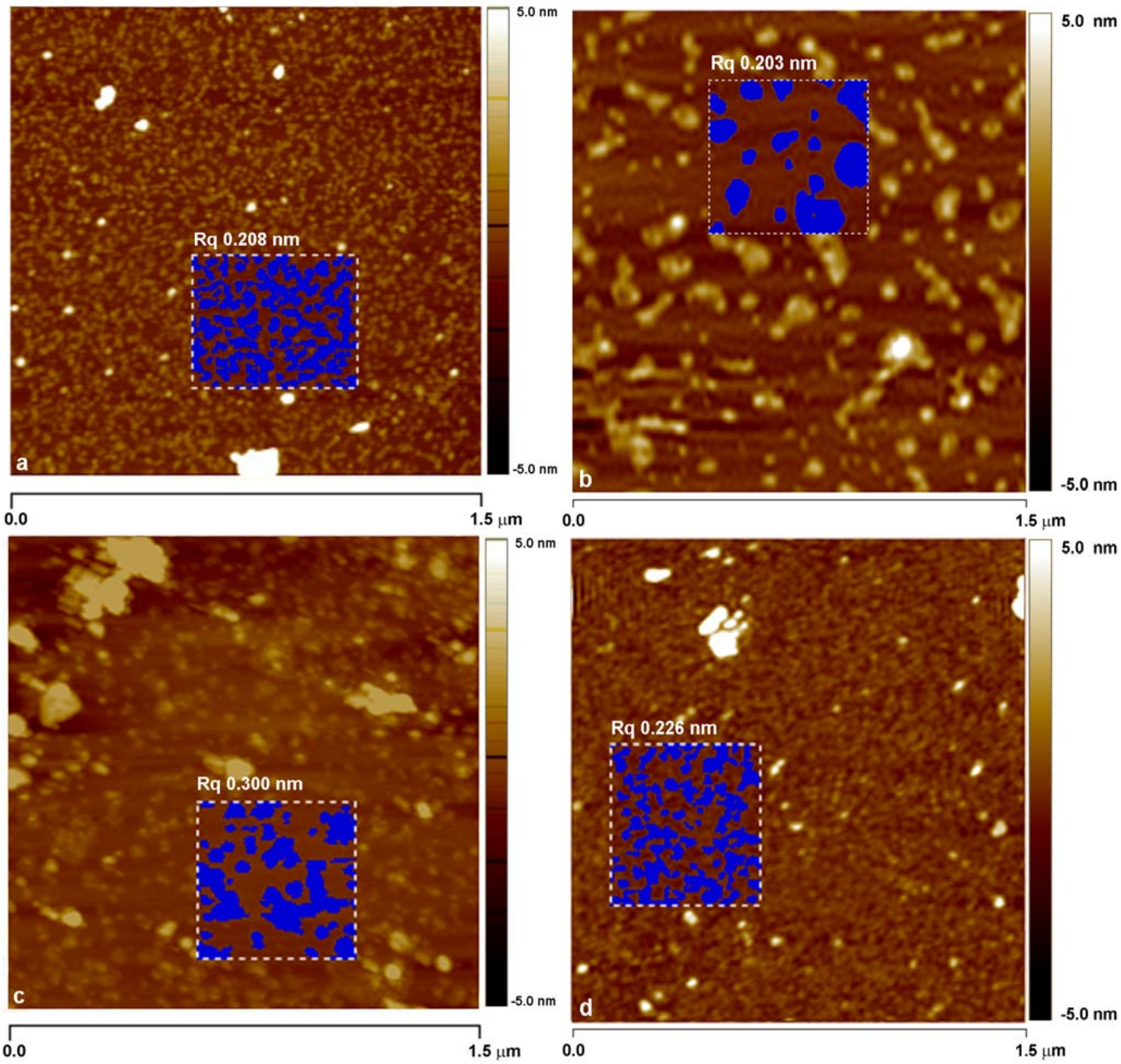

Figure S5: Analysis of surface root mean square roughness (R_q) for PSAMs of P4 coated on bare mica (a) and supports primed with (b) CA (c) NAC (d) TG.

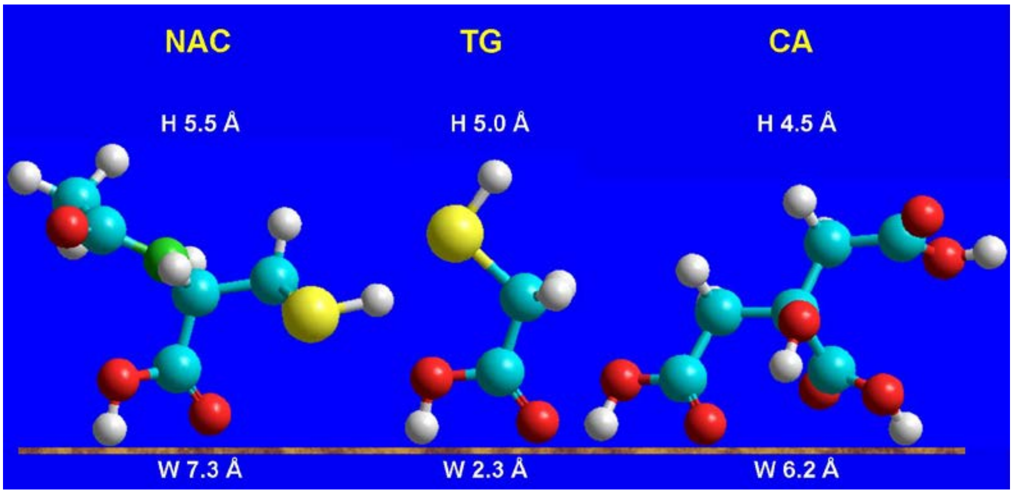

Figure S6: Structure of primers used for the modification of mica [constructed on HyperChem platform and modelled in vacuo using a molecular mechanics force field MM+ method (Polak–Ribiere/conjugate gradient optimization algorithm) and a semi-empirical PM3 method (single point energy calculations)] and their size (H – height, W – width) for a tentative orientation on mica surface (anchoring with COOH groups).

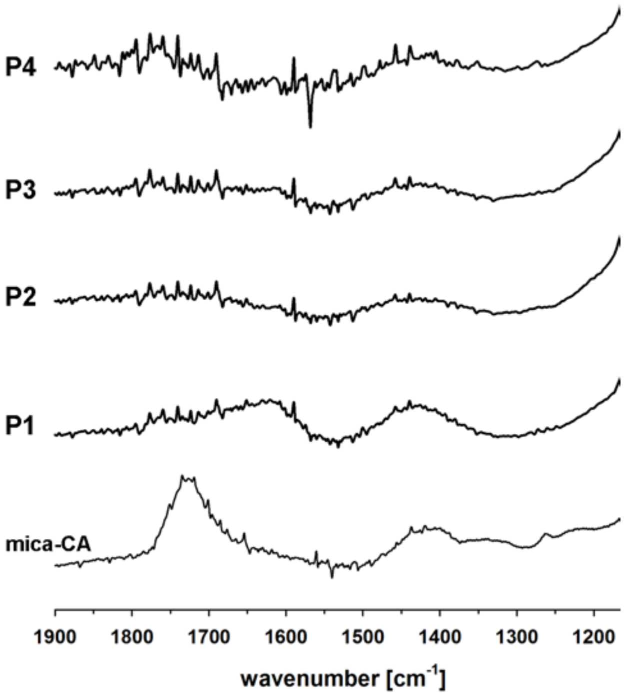

Figure S7: ATR-FTIR spectra (1900-1150 cm^{-1} region) of P1, P2, P3 and P4 adsorbed on mica-CA.

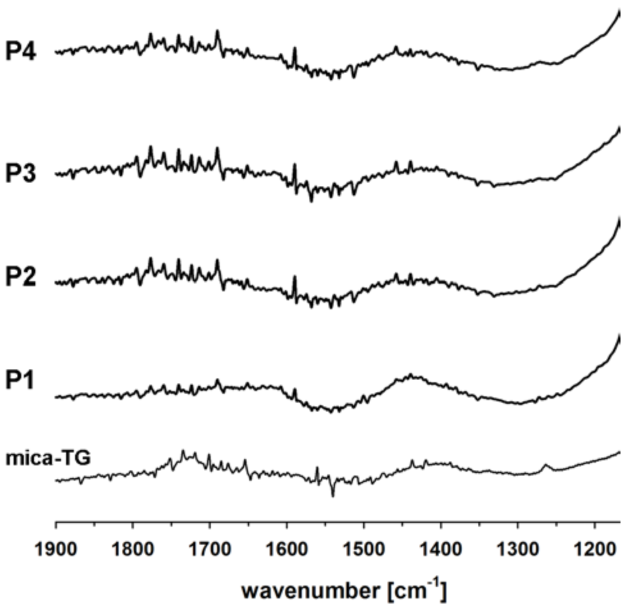

Figure S8: ATR-FTIR spectra (1900-1150 cm^{-1} region) of P1, P2, P3 and P4 adsorbed on mica-TG.

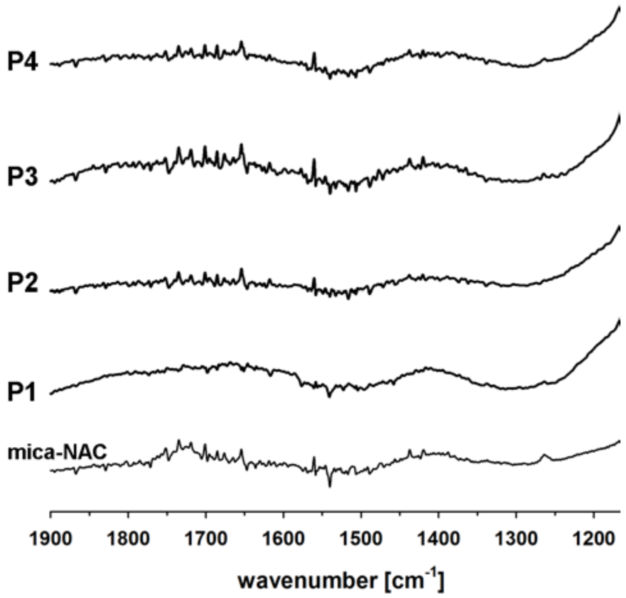

Figure S9: ATR-FTIR spectra (1900-1150 cm^{-1} region) of P1, P2, P3 and P4 adsorbed on mica-NAC.
